# Patients with spina bifida and bladder cancer. Our experience

**DOI:** 10.1186/1743-8454-7-S1-S54

**Published:** 2010-12-15

**Authors:** Esther Pagès, Lluisa Montesinos, Mar Meléndez, Susana Rodríguez, Ampar Cuxart

**Affiliations:** 1 Spina Bifida Unit. Rehabilitation Department. Vall d’Hebron Hospital. Barcelona, Spain

## Background

Patients with neurogenic bladder dysfunction due to spina bifida have been reported to be at increased risk for bladder cancer. The publications suggest that intermittent or permanent catheterization, bacteriuria, bladder calculi and bladder augmentation are a significant risk factor. We reviewed our experience with treating patients with spina bifida and bladder cancer.

## Materials and methods

Patients with spina bifida treated for bladder cancer between 1990 and 2010 were identified. Patient demographics, mode of bladder management, risk factors and presenting symptoms were recorded along with therapy, pathological findings and outcome. A review of all known published studies was made.

## Results

We found four patients with a mean age of 32.75 years old, one man and three women. Any patient had undergone bladder augmentation. Three patients used as mode of bladder management intermittent catheterization or permanent urethral catheter, and the male had used collector and had been diagnosed of repetitive bladder calculi. Abdominal pain was the first presenting symptom in two patients, hematuria in another one and the last one was diagnosed because of an ureteral obstruction. All of them had previous history of recurrent urinary tract infections. 75% patients had locally advanced stage (T3 or greater) or lymph node metastases at the time of diagnosis. Two patients had died at the time of the study.

## Conclusions

Bladder cancer should be considered on in this patient population, even in young adult women. Therefore a complete screening would be beneficial for earlier detection and improved outcomes in every spina bifida patient with hematuria or chronic infection.

